# Comparison of two protective lung ventilatory regimes on oxygenation during one-lung ventilation: a randomized controlled trial

**DOI:** 10.1186/1749-8090-5-99

**Published:** 2010-11-02

**Authors:** Félix R Montes, Daniel F Pardo, Hernán Charrís, Luis J Tellez, Juan C Garzón, Camilo Osorio

**Affiliations:** 1 Department of Anesthesiology. Fundación CardioInfantil - Instituto de Cardiología. Calle 163 A # 13B - 60. Bogotá, Colombia, South América; 2 Department of Thoracic Surgery. Fundación CardioInfantil - Instituto de Cardiología. Calle 163 A # 13B - 60. Bogotá, Colombia, South América

## Abstract

**Background:**

The efficacy of protective ventilation in acute lung injury has validated its use in the operating room for patients undergoing thoracic surgery with one-lung ventilation (OLV). The purpose of this study was to investigate the effects of two different modes of ventilation using low tidal volumes: pressure controlled ventilation (PCV) vs. volume controlled ventilation (VCV) on oxygenation and airway pressures during OLV.

**Methods:**

We studied 41 patients scheduled for thoracoscopy surgery. After initial two-lung ventilation with VCV patients were randomly assigned to one of two groups. In one group OLV was started with VCV (tidal volume 6 mL/kg, PEEP 5) and after 30 minutes ventilation was switched to PCV (inspiratory pressure to provide a tidal volume of 6 mL/kg, PEEP 5) for the same time period. In the second group, ventilation modes were performed in reverse order. Airway pressures and blood gases were obtained at the end of each ventilatory mode.

**Results:**

PaO_2_, PaCO_2 _and alveolar-arterial oxygen difference did not differ between PCV and VCV. Peak airway pressure was significantly lower in PCV compared with VCV (19.9 ± 3.8 cmH_2_O vs 23.1 ± 4.3 cmH_2_O; p < 0.001) without any significant differences in mean and plateau pressures.

**Conclusions:**

In patients with good preoperative pulmonary function undergoing thoracoscopy surgery, the use of a protective lung ventilation strategy with VCV or PCV does not affect the oxygenation. PCV was associated with lower peak airway pressures.

## Introduction

Anesthesia for thoracic surgery routinely involves one lung ventilation (OLV) to provide optimum surgical operating conditions and to isolate and protect the lungs during the procedure. Unfortunately, this practice may associate with an important impairment in gas exchange, particularly in patients with previous lung disease [1].

OLV traditionally has been performed with tidal volumes (V_T_) that are equal to those being used on two lung ventilation (TLV) [2]. Over the past decades, V_T _used by clinicians have progressively decreased from more than 12-15 ml/kg to less than 9 ml/kg actual body weight [3-6]. This practice is based on several studies that showed that mechanical ventilation using V_T _of no more than 6 ml/kg resulted in reduction of systemic inflammatory markers, increased ventilator-free days, and reduction in mortality when compared with V_T _of 12 ml/kg in patients with acute lung injury (ALI) and acute respiratory stress syndrome [7,8]. The reduction of V_T _has been recommended in patients without pulmonary pathology at the onset of mechanical ventilation [9].

The use of low V_T _has been also recommended in patients during OLV [10]. Recent studies have suggested that low V_T _during OLV can be associated with a decreased incidence of complications [11-13]. However the effects of low V_T _on oxygenation in patients undergoing thoracic surgery with OLV have been less examined. In the operating room, volume controlled ventilation (VCV) is commonly used and it has become the dominant ventilator mode. However, the mechanical characteristics of pressure controlled ventilation (PVC) are thought to allow more homogeneous distribution of ventilation and improved ventilation-perfusion matching [14]. The aim of this study is to evaluate the impact of two currently used protective lung ventilation strategies on oxygenation during OLV in patients undergoing thoracic surgery.

## Patients and Methods

After approval by the Fundación Cardio Infantil-Instituto de Cardiología ethics committee and after obtaining written informed consent from each individual, we enrolled into the study 41 patients undergoing elective thoracic surgery requiring at least 1 hour of OLV. All patients were ASA physical status I-III and aged between 18 and 75 years. Patients with a documented history of uncompensated cardiac, hepatic o renal disease were excluded from the study. All patients underwent arterial blood gases and lung spirometry prior to surgery.

Upon arrival to the operating room, patients were monitored with electrocardiogram and SpO_2_. A 14-gauge IV catheter was inserted in an upper extremity vein and a 20-gauge catheter was inserted in a radial artery to permit continuous recording of arterial pressure. After preoxygenation, anesthesia was induced with remifentanil 0.2 μg/kg/min, propofol 2 mg/kg, and cisatracurium 0.15 mg/kg. Anesthesia was maintained with a continuous infusion of remifentanil 0.1 μg/kg/min, propofol 100 μg/kg/min, and supplemental cisatracurium. Clinical signs of light anesthesia characterized by hemodynamic responses to surgical stimulation [median arterial blood pressure (MAP) > 20% of the preinduction baseline values and/or heart rate (HR) > 90 bpm], somatic (patient movement, eye opening) or autonomic (lacrimation, sweating) responses were treated with boluses of remifentanil 0.5 μg/kg followed by 50% increments in the infusion rate. A minimum time of 1 minute was required between infusion rate increases. Excessive depth of anesthesia judged by hypotension (MAP < 20% of the preinduction baseline) and/or bradycardia (HR < 40 bpm) was treated by a 50% decrement in the remifentanil infusion rate. If this treatment proved inadequate, IV etilefrine (for hypotension) or atropine (for bradycardia) was administered. The propofol infusion was unchanged. No volatile anesthetics were used. The trachea was intubated with a double lumen tube (Mallinckrodt-BroncoCath, Tyco Health Care, Pleasanton, CA) no. 37 for male and no. 35 for female patients. Left double-lumen tubes were chosen as long as there was no contraindication. The position of the tube was confirmed by auscultation and fiberoptic bronchoscopy before and after turning the patient to the lateral decubitus position.

Initially, TLV with VCV was performed in all patients using a FIO_2 _of 1.0, a V_T _of 9 mL/kg, and a ventilator rate of 12/min, then adjusted to maintain end-tidal carbon dioxide tension (E_T_CO_2_) of 25 to 30 mmHg (Servo 900C; Siemens, Solna, Sweden) [Normal arterial oxygen and carbon dioxide tension in Bogota are 60 ± 3 and 30 ± 3 mm Hg respectively (8700 ft or 2600 m above sea level)]. The inspiratory time and the end-inspiratory pause time were adjusted as 25% and 10% respectively, and it was unchanged during all the study. No external positive end expiratory pressure (PEEP) was applied during this period. Prior initiation of OLV, patients were randomly assigned, according to a computer-generated random number table, to one of two groups. Group A: OLV was started by VCV (OLV-VCV) using a V_T _of 6 mL/kg, PEEP of 5 cm H_2_O, and the ventilator rate adjusted to maintain a E_T_CO_2 _of 25 to 30 mmHg. After 30 min PCV (decelerating inspiratory flow) was started with a FIO_2 _of 1.0, PEEP of 5 cm H_2_O, a peak airway pressure adjusted to obtain the same V_T _as during VCV, and a ventilator frequency adjusted to keep E_T_CO_2 _of 25 to 30 mmHg. Group B: PCV was initiated with a peak airway pressure that provided a V_T _of 6 mL/kg, PEEP of 5 cm H_2_O, and a ventilator rate adjusted to maintain E_T_CO_2 _of 25 to 30 mmHg. After 30 min the ventilator was changed to VCV with a V_T _6 mL/kg, PEEP of 5 cm H_2_O, and the ventilator frequency adjusted to maintain a E_T_CO_2 _of 25 to 30 mmHg.

Blood gas analysis, hemodynamic measurements, peak inspiratory pressure (Ppeak), mean inpiratory (Pmean), plateau inspiratory pressure (Pplateau), and expired V_T _were measured and recorded at four stages: (1) During TLV using VCV prior the beginning of OLV; (2) During OLV 30 min after initiation of the first ventilation mode; (3) During OLV 30 min after the second ventilator mode; and (4) End of surgery: 30 min after reestablishing TLV with VCV. During the measurement period surgical manipulation of the lung was not allowed.

A power analysis based on a previous study [15] revealed a total sample size of 38 patients was required to achieve a power of 80% and an α of 0.05 for detection of 40 mmHg difference in the PaO_2 _value. Student's t test and ANOVA were used to determine the significance of normally distributed parametric values. Categorical variables were tested using χ2 test or, when appropriate, Fisher's exact test. Statistical significance was accepted at p < 0.05.

## Results

Forty-one patients were enrolled into the study. There were no significant differences between the two groups in demographic characteristics, type of surgical procedure performed or pre-operative lung function test (table 1). No patient was excluded from the study due to any preoperative o intraoperative criteria, and in all patients left-double lumen tubes were used.

**Table 1:** Demographic characteristics of patients

	Group A	Group B	*P*
	*n *= 20	*n *= 21	
Age (yrs)	56.1 ± 17	59.1 ± 16	0.39
Weight (kg)	65.0 ± 11.9	63.0 ± 11.4	0.59
Height (cm)	161.5 ± 12.2	159.5 ± 11.0	0.60
Sex (F/M)	11/8	14/7	0.75
Side of surgery (R/L)	11/9	16/6	0.21
Type of surgery			
Lobectomy	6	4	
Wedge resections	8	12	
Medistinal tumor	4	3	
Other	2	2	
Preoperative PaO_2 _(mmHg)	61,2 ± 4,8	60,2 ± 4,1	0,43
Preoperative PaCO_2 _(mmHg)	32,3 ± 3,5	31,6 ± 2,6	0,23
Preoperative FEV_1 _(% predicted)	95,4 ± 20,8	86,9 ± 17,6	0,24
Preoperative FVC (% Predicted)	96,6 ± 20,3	88,6 ± 14,5	0,23
			

Data are shown as mean ± SD.

FEV_1 _= forced expiratory volume in 1 second; FVC = forced vital capacity; PaO_2 _= arterial blood oxygen tension; PaCO_2 _= arterial blood carbon dioxide tension.

The beginning of OLV with either VCV or PCV produced a significant increase in mean (p < 0.001), and plateau (p < 0.01) airway pressures; the Ppeak was significantly higher in VCV patients (p = 0.001) but not in PCV patients (p = 0.53) compared with the initial TLV. As expected, the PaO_2 _with any mode of OLV was significantly lower compared to TLV (p < 0.001) and increased to a similar level after switching again to TLV. Comparison of the OLV-VCV and OLV-PCV showed a significant difference in Ppeak (p = 0.003) without differences in Pmean, Pplateau PaO_2_, and PaCO_2_, (Table 2). The sequence of OLV did not influence the airway pressures or blood gases values.

**Table 2:** Intraoperative Variables

	TLV-VCV	OLV-VCV	OLV-PCV	End of Surgery
	*n *= 41	*n *= 41	*n *= 41	*n = 41*
V_T _(mL)	562 ± 109	377 ± 80^a^	386 ± 82^a^	524 ± 149
Ppeak (cmH_2_O)	18.7 ± 4.3	23.1 ± 4.3^a^	19.9 ± 3.8^c^	17.4 ± 3.5
Pmean (cmH_2_O)	5.6 ± 3.8	9.6 ± 1^a^	9.5 ± 1.3^a^	5.5 ± 1.9
Pplateau (cmH_2_O)	14.2 ± 3.8	16.8 ± 2.5^a^	16 ± 2.7^b^	13 ± 2.7
pH	7.45 ± 0.05	7.42 ± 0.04	7.43 ± 0.04	7.44 ± 0.05
PaO_2 _(mmHg)	277 ± 97	101 ± 52^a^	111 ± 56^a^	293 ± 91
PaCO_2 _(mmHg)	29.2 ± 4.3	32.4 ± 3.7	31.6 ± 3.9	29.4 ± 4.5
SaO_2 _(%)	99.3 ± 1	95.9 ± 3.2^a^	96.1 ± 3.4^a^	99.4 ± 1.1
A-aO_2 _D	198 ± 95	372 ± 51^a^	363 ± 56^a^	184 ± 89

Data are shown as mean ± SD.

A-aO_2_D = Alveolar-arterial oxygen difference; OLV = One-lung ventilation; PaCO_2 _= arterial carbon dioxide tension; PaO2 = arterial oxygen tension; PCV = Pressure controlled ventilation; Pmean = mean inspiratory pressure; Ppeak = peak inspiratory pressure; Pplateau = plateau inspiratory pressure; SaO2 = arterial oxygen saturation; TLV = Two-lung ventilation; VCV = Volume controlled ventilation; VT = Tidal volume; ^a ^*p *< 0.001 compared with TLV-VCV; ^b ^*p *< 0.01 compared with TLV-VCV; ^c ^*p *< 0.01 compared with OLV-VCV

## Discussion

The ventilator strategy recommended to reduce the incidence of ALI in patients undergoing thoracic surgery is to use lower Vt (5-7 mL/kg) with moderate amounts of PEEP (5-6 cmH_2_O) [10,16]. The present study suggests that using any of the common available ventilator modes, VCV or PCV with a "lung protective" approach, results in similar effects on oxygenation and gas exchange.

VCV has been considered the traditional or conventional approach to mechanical ventilation of patients undergoing thoracic surgery and OLV. However, in recent years PCV has gained renew interest due to its potential advantages [2,17,18]. VCV uses a constant inspired flow (square wave), creating a progressive increase of airway pressure toward the peak inspiratory pressure, which is reached as the full tidal volume has been delivered. Unlike VCV, PVC ventilator mode produces appropriate flow to rapidly reach and maintain the set inspiratory pressure (square pressure waveform). The resultant respiratory flow is usually decelerating, minimizing peak airway pressures, and theoretically resulting in more homogeneous distribution of Vt, improvement in static and dynamic lung compliance, better oxygenation and dead space ventilation [19].

The literature concerning the comparative effects of PCV and VCV on intraoperative arterial oxygenation during OLV has produced inconsistent results. Tugrul *et al *found a statistically significant decrease in Ppeak and Pplateau and improved oxygenation and intrapulmonary shunt with PVC compared to VCV in patients undergoing thoracotomy using a Vt of 10 mL/kg during TLV and OLV. The findings were more relevant in subjects who had poor preoperative lung function [17]. In a subsequent study, Senturk *et al *showed that PCV with a PEEP of 4 cmH_2_O was associated with an improvement in oxygenation compared to VCV and zero PEEP [18]. However, other groups have not been able to reproduce the oxygenation benefit using PVC during OLV [15,20,21]. It is important to point out that all those studies used a Vt between 8-10 mL/kg which is higher than the 5-7 mL/kg recommended for protective ventilation during OLV. Although using lower Vt still lacks a clear demonstration of clinical outcome benefits, a growing body of scientific evidence indicates that traditional Vt of around 10 mL/kg maybe injurious in the healthy lungs. Schilling *et al *reported reduced alveolar concentrations of TNF-α in patients undergoing thoracotomy ventilated with small vs. large Vt (5 vs. 10 mL/kg) [13]. Consistent with those results, Michelet *et al *reported a decreased proinflammatory response, improved oxygenation index and earlier extubation in patients undergoing esophagectomy who received low Vt (5 mL/kg) with a PEEP level of 5 cmH_2_O compared with subjects receiving Vt of 10 mL/kg and zero PEEP [12].

Exposure to an elevated inspiratory pressure during OLV has been identified as strong predictor of ALI in patients undergoing thoracic surgery and during TLV in high-risk elective surgeries [22-24]. However, it is unclear which of the commonly measured pressures is more relevant in the development of complications. The Ppeak is a reflection of the dynamic compliance of the respiratory system and depends on issues such as tidal volume, inspiratory time, endotracheal size, and bronchospasm. In contrast, Pplateau relates to the static compliance of the respiratory system (ie, chest wall and lung compliance) and is considered a better reflection of alveolar pressure. On the other hand, Pmean correlates with alveolar ventilation and gas oxygenation [25]. Van der Werf and colleagues analyzed 197 consecutive patients who underwent lung resection and found that high Ppeak was associated with the development of postpneumonectomy pulmonary edema (relative risk, 3.0; 95% confidence interval, 1.2 to 7.3) [23]. Recently, a prospective case control study found that mildly increased Ppeak -21 cm H_2_O- was likely to contribute to the development of ALI on patients undergoing major surgery (OR 1.07; 95% CI 1.02 to 1.15) [24]. In addition, a study looking at risk factors for ALI after thoracic surgery in lung cancer patients, found that excessive Pplateau -29 cm H_2_O- were likely to have contributed to the development of ALI in these patients (OR 3.5; 95% CI 1.7-8.4) [22].

In our study we found differences in Ppeak, while the Pplateau were similar in both groups. However, the pressure values in PCV and VCV groups were below those currently recommended in this type of surgery: Ppeak less than 35 cm H_2_O and Pplateau less than 25 cm H_2_O [10,26]. Our results are consistent with those of Roze *et al *who compared airway pressure in the breathing circuit with that in the dependent lung bronchus during VCV followed by PCV. These authors observed that PCV reduced both circuit pressure and bronchial pressure but the decrease in Ppeak was significantly higher in the circuit. They found a small reduction in bronchial airway pressure that is probably not clinically significant [27]. A limitation of this study should be mentioned. The patients involved had near-normal pulmonary function; thus, these results may not extrapolate to sicker patients with compromised pulmonary function. Some authors believe that pressure limitation obtained with PCV may be useful in certain populations (i.e. obstructive lung disease) where decelerating waveforms may diminish the risk of barotrauma and decrease the likelihood of unintentional hypoventilation [28].

In conclusion, in patients without severe lung disease undergoing thoracic surgery with OLV, lung-protective strategies using "low Vt" combined with PEEP is safe and effective. The pressure-controlled mode of ventilation (vs. volume-controlled mode) decreases peak airway pressure maintaining similar blood oxygenation indices.

## Competing interests

The authors declare that they have no competing interest.

## Authors' contributions

FRM: Study design, development of methodology, collection and analysis of data, writing the manuscript. DFP: Study design, collection, analysis and interpretation of data. HC: Study design, development of methodology, supervision. LJT: Study design, collection and analysis of data. JCG: Study design, collection and analysis of data. CO: Study design, collection and analysis of data. All authors have read and approved the final manuscript.
